# Three-dimensional in vitro model of bone metastases of neuroblastoma as a tool for pharmacological evaluations

**DOI:** 10.7150/ntno.85439

**Published:** 2024-01-01

**Authors:** Sanja Aveic, Max Seidelmann, Roswitha Davtalab, Diana Corallo, Michael Vogt, Stephan Rütten, Horst Fischer

**Affiliations:** 1 Department of Dental Materials and Biomaterials Research, RWTH Aachen University Hospital, Pauwelsstrasse 30, 52074 Aachen, Germany.; 2 Laboratory of Target Discovery and Biology of Neuroblastoma, Istituto di Ricerca Pediatrica Fondazione Città della Speranza, Corso Stati Uniti 4, 35127 Padova, Italy.; 3 Interdisciplinary Center for Clinical Research, RWTH Aachen University Hospital, Pauwelsstrasse 30, 52074 Aachen, Germany.; 4 Electron Microscopy Facility, Institute of Pathology, RWTH Aachen University Hospital, Pauwelsstrasse 30, 52074 Aachen, Germany.

**Keywords:** 3D geometry, bone metastatic niche, drug screening, neuroblastoma

## Abstract

*In vitro* metastatic models are foreseen to introduce a breakthrough in the field of preclinical screening of more functional small-molecule pharmaceuticals and biologics. To achieve this goal, the complexity of current *in vitro* systems requests an appropriate upgrade to approach the three-dimensional (3D) *in vivo* metastatic disease. Here, we explored the potential of our 3D β-tricalcium phosphate (β-TCP) model of neuroblastoma bone metastasis for drug toxicity assessment. Tailor-made scaffolds with interconnected channels were produced by combining 3D printing and slip casting method. The organization of neuroblastoma cells into a mesenchymal stromal cell (MSC) network, cultured under bioactive conditions provided by β-TCP, was monitored by two-photon microscopy. Deposition of extracellular matrix protein Collagen I by MSCs and persistent growth of tumor cells confirmed the cell-supportive performance of our 3D model. When different neuroblastoma cells were treated with conventional chemotherapeutics, the β-TCP model provided the necessary reproducibility and accuracy of experimental readouts. Drug efficacy evaluation was done for 3D and 2D cell cultures, highlighting the need for a higher dose of chemotherapeutics under 3D conditions to achieve the expected cytotoxicity in tumor cells. Our results confirm the importance of 3D geometry in driving native connectivity between nonmalignant and tumor cells and sustain β-TCP scaffolds as a reliable and affordable drug screening platform for use in the early stages of drug discovery.

## 1. Introduction

Bioceramics based on calcium phosphates have been widely used in bone tissue engineering due to their excellent bioactivity, osteoinductive, and osseointegrative properties. In fact, sintering of tricalcium phosphate (β-TCP) scaffolds allows the generation of well-defined porous structures that sustain growth and effective attachment of various cell types, including normal (stromal, endothelial) and neoplastic cells.^1^,^2^ The presence of Ca2+ ions in these bioceramics affects the cell behavior and triggers differentiation of mesenchymal stromal cells (MSC) towards bone-progenitors.^3^-^5^ Nowadays, the interest in β-TCP ceramics has increased among the scientific community that is seeking biologically relevant three-dimensional (3D) disease models of the bone.^6^-^8^ They have proven to be a remarkable material for the reproduction of 3D scaffolds with new functions, using different methods including slip casting, tape casting or extrusion.^9^ Moreover, the potential of 3D *in vitro* models is particularly promising for accelerated drug discovery and more effective treatment screenings.^10^,^11^ This expectation is reasonable due to the fact that the 3D cell models offer increased complexity in comparison to traditional two-dimensional (2D) platforms, and thus more closely approximate the cell organization and extracellular matrix (ECM) composition of an *in vivo* system.^12^,^13^ Each of these characteristics play a determining role in defining the distribution of an administered drug and in orchestrating the type or tumor cell reaction to it.^14^ In the case of bone and bone marrow metastases, a threatening consequence of cancer progression in many types of solid tumors, 3D *in vitro* bone models can offer a vivid facsimile of the *in vivo* malignant microenvironment.^15^-^17^ Likewise, more complex disease models are expected to provide reliable data that can better approximate the actual native compartment of the bone or bone marrow metastatic disease in terms of biological mechanisms of action.^18^ Consequently, they can be a valuable source for pharmacological discovery and for the evaluation of more effective anti-metastatic drugs.

Among other malignancies, bone metastasis of neuroblastoma are particularly aggressive and determine a poor prognosis for patients.^19^ In fact, neuroblastoma is the most lethal extra-cranial solid tumor in patients of preschool age and remains one of the most enigmatic and aggressive malignancies in pediatric oncology.^20^ More than 50% of patients affected by neuroblastoma are classified as high-risk, and the prognosis attributed to them is reserved due to a limited efficacy of currently available therapies.^21^ The tumors found in high-risk patients are particularly refractory to chemotherapy with frequent dissemination of tumor cells into the bone and bone marrow.^22^ For this group of patients there is an urgent need for therapy reinforcement through innovative and tailored (adjuvant) treatment options. However, the introduction of new disease models, which are largely lacking today in pediatric solid malignancies where the amount of tumor material is very limited and often insufficient for comprehensive biological studies, could help to develop more reliable drug candidates in a shorter time.

In this study, we explored the versatility of our well-established 3D β-TCP scaffolds with interconnected channel structures of defined size as a platform for drug efficacy evaluations. We explored the influence of chemotherapeutic agents on a co-culture of stromal and neuroblastoma tumor cells. Based on cell biology data, and in relation to conventional 2D experimental settings, we determined the strength of our 3D scaffolds for preclinical pharmacological evaluations and endorsed their applicability for future screenings of the anti-metastatic compounds.

## 2. Material and Methods

### 2.1. Cell lines and primary cells

Human mesenchymal stromal cells (MSC) were obtained by flushing the bone marrow from a hip obtained during surgical treatments at the Department of Orthopedics of the RWTH Aachen University Hospital, approved by the ethics committee of the Faculty of Medicine of RWTH Aachen University (EK 300/13). The MSC were cultured in basic Mesenpan medium supplemented with 2 % FCS, 1 % penicillin/streptomycin/glutamine and 1 % MSC growth supplement (all from PAN Biotech, Aidenbach, Germany), and used until passage number 6. Media was changed every 2-3 days. Three different donors were considered. Neuroblastoma cancer cells (SH-SY5Y and SK-N-BE2) originating from a bone marrow metastasis were purchased from DSMZ (Deutsche Sammlung von Mikroorganismen und Zellkuturen, Braunschweig) and ATCC (American Type Culture Collection), respectively. The SH-SY5Y, with or without stably expressing GFP-protein,^23^ and SK-N-BE2 cells were grown in RPMI medium (Gibco) supplemented with 10 % FCS and 100 U/ml penicillin/streptomycin (all from PAN Biotech). Media was changed every 2-3 days. The cell seeding inside the interconnected channels was multistep and included: i) pre-equilibration of the scaffolds in the Mesenpan medium for 30 minutes prior to seeding; ii) addition of 30,000 MSC / 30 µl of Mesenpan medium and growth for 3 weeks; and iii) addition of 10,000 SH-SY5Y cells and growth for one more week in a Mesenpan medium. The optimal cell number and growth conditions have been previously adjusted.^8^ Total cell number was determined using trypan blue on a Countess^TM^ automatic cell counter (Invitrogen, Darmstadt, Germany). A flow cytometry procedure for measuring GFP positive events was performed in triplicates as described elsewhere.^18^

### 2.2. Scaffold fabrication

The 3D scaffolds with 500 μm interconnected micro-channels were prepared by printing wax negative molds starting from customized Computer Aided Design (CAD) models. The molds consisted of a wax model (diameter 8.5 mm, height 4.5 mm) with defined pore geometry of interconnecting channels (diameter of the channels 500 µm) and a separately printed baseplate. The wax molds were printed by 3D-printer (T76+, Solidscape, New Hampshire, UK) consisting of two different components: supporting wax (magenta; Indura®Fill, Solidscape, New Hampshire, USA) and construction wax (grey; Indura®Cast, Supplementary Figure 1a), allowing the development of the channeled 3D structures.

The molds were printed layer-by-layer using drop on demand technology. After printing, the supporting wax was solved in petroleum (Merck 1.09718, Merck, Darmstadt, Germany) to get the definite wax mold. The mold was fixed to the base (Supplementary Figure 1b, 1c). After printing, the molds were processed as described previously 8, and filled with β-TCP slurry (Supplementary Figure 1d) composed of 31.25 % organic solvent (Contraspun, Optapix PAF, and Dolapix CE64 in H_2_O) and 68.75 % of inorganic β-TCP powder (Budenheim Pharmaceutical Technology, Germany). Sintering of β-TCP ceramics was done after drying them overnight at room temperature. Sintering temperature for β-TCP ceramics was held at 1100°C for 3 hours.^8^ Alumina (Al_2_O_3_) was used as a bioinert counterpart to β-TCP scaffolds to exclude the influence of Ca++ on MSC behavior.^24^ Alumina slurry was produced as a suspension containing 69.84 % alumina powder (CT3000 LS SG, Almantis GmbH, Frankfurt, Germany), 28.52 % H_2_O, 1.05 % polyethylenglycol 400 (Carl Roth, Karlsruhe, Germany), 0.01 % Contraspun and 0.58% Dolapix CE64 (both from Schirmer & Schwarz Chemische Fabriken, Lahnstein, Germany). The sintering temperature at 1500°C (heating rate 3 K/minute) was reached and maintained for 2 hours (Supplementary Figure 1e). For the *in vitro* cell culture use, the scaffolds were sterilized using autoclave (Systec DE 23, Systec, Linden, Germany) and dried in the furnace (Heraeus T 5028, Heraeus, Hanau, Germany) at 200°C for 20 minutes.

### 2.3. Manufacturing quality control

The 3D scaffolds were scanned by the micro-CT (µCT; Supplementary Video 1a) using SkyScan 1172, (Kontich, Belgium) to control the quality of the produced scaffolds and the presence of voids due to the slip-casting technique. The images were analyzed using the open-source software Fiji ImageJ (Rayn Rasband, National Institutes of Health, Maryland, USA).^25^ Additionally, the β-TCP material was analyzed to assure a properly working sintering process by using X-ray diffraction (Bruker Advanced D8, Bruker, Billerica, USA)**.** The microstructure of the scaffolds was assessed applying scanning electron microscopy (SEM) (FEIESEMXL30FEG, Philips, Eindhoven, Netherlands) and using an electron beam (EDS, FalconGenesis; EDAX, Mahwah, NJ) as described later. Analysis was done using Fiji ImageJ software.

### 2.4. Cell viability and drug treatments

Cell viability was recorded applying CCK-8 indirect viability assay (CCK-8, Dojindo Laboratories, Kumamoto, Japan). The kit was used according to the producer's manual and the absorbance was measured at 450 nm wavelength in a microplate reader (Spectramax M2, Molecular devices, San José, USA). A treatment with 5 µM and 10 µM of Etoposide (VP16, Sigma-Aldrich) or 5 µM and 10 µM of Cisplatin (Cispl, Sigma-Aldrich) was done 3 days after seeding of neuroblastoma cells for a total of 96 hours. The results were compared with conventional 2D culture system. As controls (CTRL), the cells treated with vehicle (DMSO) were considered.

### 2.5. Microscopic cell analysis

Cell growth inside the interconnected channels was monitored under light microscope (Zeiss Promovert, Zeiss, Oberkochen, Germany). After 28 days, the samples were processed for further imaging with either scanning electron (SEM) or two-photon microscopes. The SEM was also used to analyze the cell distribution inside the channels. Before imaging, the samples were fixed in 3 % glutaraldehyde (Agarscientific, Wetzlar, Germany), washed in 0.1 M Soerensen's Phosphate Buffer (Merck, Darmstadt, Germany) for 15 minutes, dehydrated in an ascending dilution of acetone, and then dried using a critical point drying method in liquid CO_2_ (CriticalPointDryer, Polaron, QuorumTechnologiesLtd, Ashford, England). Subsequently, the samples were coated with 10 nm gold/palladium film (Sputter Coater EM SCD500, Leica, Wetzlar, Germany), microscopy was performed in a high vacuum environment (ESEM XL30 FEG, FEI, Eindhoven, The Netherlands), and 10kV acceleration voltage as described before.^8^ Analysis of the cell infiltration and cell interaction inside the channels was inspected using a two-photon microscope (FV1000MPE, Olympus, Tokyo, Japan) mounted with a 25x NA 1.05 water dipping objective. Previously, the samples had been fixed using 4 % formaldehyde for 3 hours as formerly described.^8^ Afterward, they were washed twice with 1 x PBS for 5 minutes. The cell membrane was permeabilized with 0.25 % of Triton X-100 (Sigma Aldrich, St. Louis, USA) prepared in 5 % BSA blocking solution (Sigma Aldrich, St. Louis, USA) for 30 minutes. For labelling F-actin, Collagen I and cleaved PARP protein, the samples were incubated overnight at 4°C with phalloidin-TRITC (1:250, Millipore, Burlington, USA), anti-Collagen 1 primary antibody (1:50, Abcam, Cambridge, UK) or anti-cleaved PARP1 (1:300, SC Biotechnology, USA) diluted in 3 % BSA solution. The samples incubated with anti-Collagen 1 or anti-cleaved PARP1 were then stained with the respective secondary antibody Alexa Flour 594 (1:1000, Abcam, Cambridge, UK) diluted in 3 % BSA solution for 1 hour at room temperature. DAPI (Thermo Fisher; 1:10,000) was used for nuclear staining. Images were analyzed using Fiji ImageJ software.

### 2.6. Statistical analysis

The results are presented as a mean value ± SD of at least three independent experiments.

Statistics were performed using one-way ANOVA followed by Tukey's Test (GraphPad Prism 7 software). The differences were considered significant for *p < 0.05, **p < 0.01, and ***p < 0.001, ****p < 0.0001.

## 3. Results

### 3.1. Scaffold portrayal

**Figure 1:**
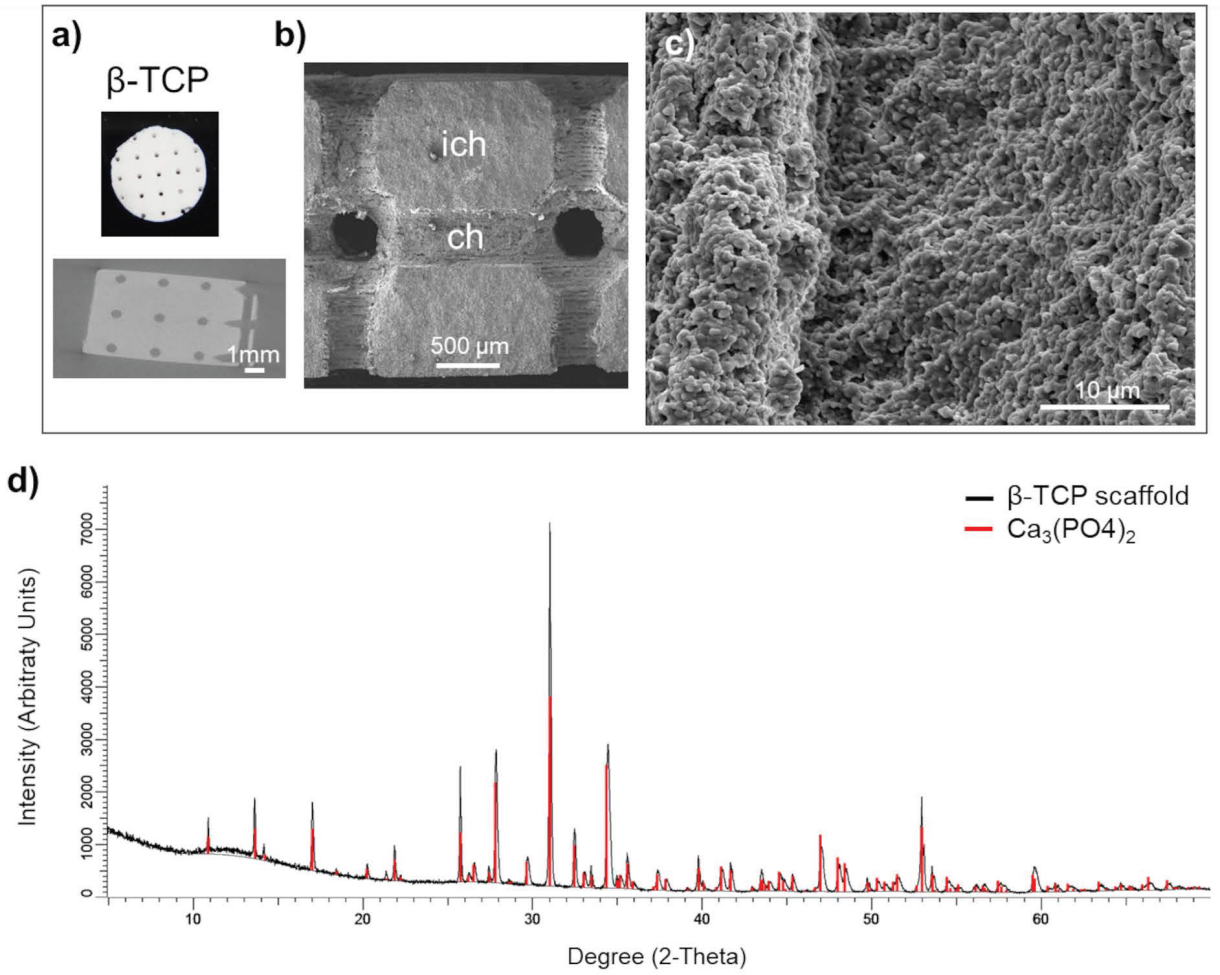
** Scaffold characterization:** a) Macrostructure and a μCT segment of the scaffold with the position of the circular (interconnected) channels of 500 μm in diameter. Scale bar, 1000 μm. b) SEM micrograph the channel (ch) surface and the inter-channel (ich) structure of β-TCP scaffold. Scale bar, 500 μm. c) Surface microstructure of β-TCP channels. d) X-ray diffraction of control β-TCP sample (red) and produced β-TCP scaffolds (black); x-axis shows the count of reflected electrons, y-axis shows the degree of the reflected electrons.

Manufacturing protocol assured a macroscopic uniformity of the beta tricalcium phosphate (β-TCP) scaffolds (Figure 1a, upper panel). The scaffolds preserved the interconnecting channel network of 500 μm in diameter (Figure 1a, lower panel). Microstructure of β-TCP (Figure 1b, 1c) scaffolds obtained after sintering process were further validated by SEM confirming expected porosity of β-TCP.^26^ The X-ray diffraction of the control sample (red line) and manufactured β-TCP scaffold (black line) implied that the analyzed product is equivalent to the control (Figure 1d).

### 3.2. 3D culturing of stromal cells in β-TCP scaffolds

**Figure 2:**
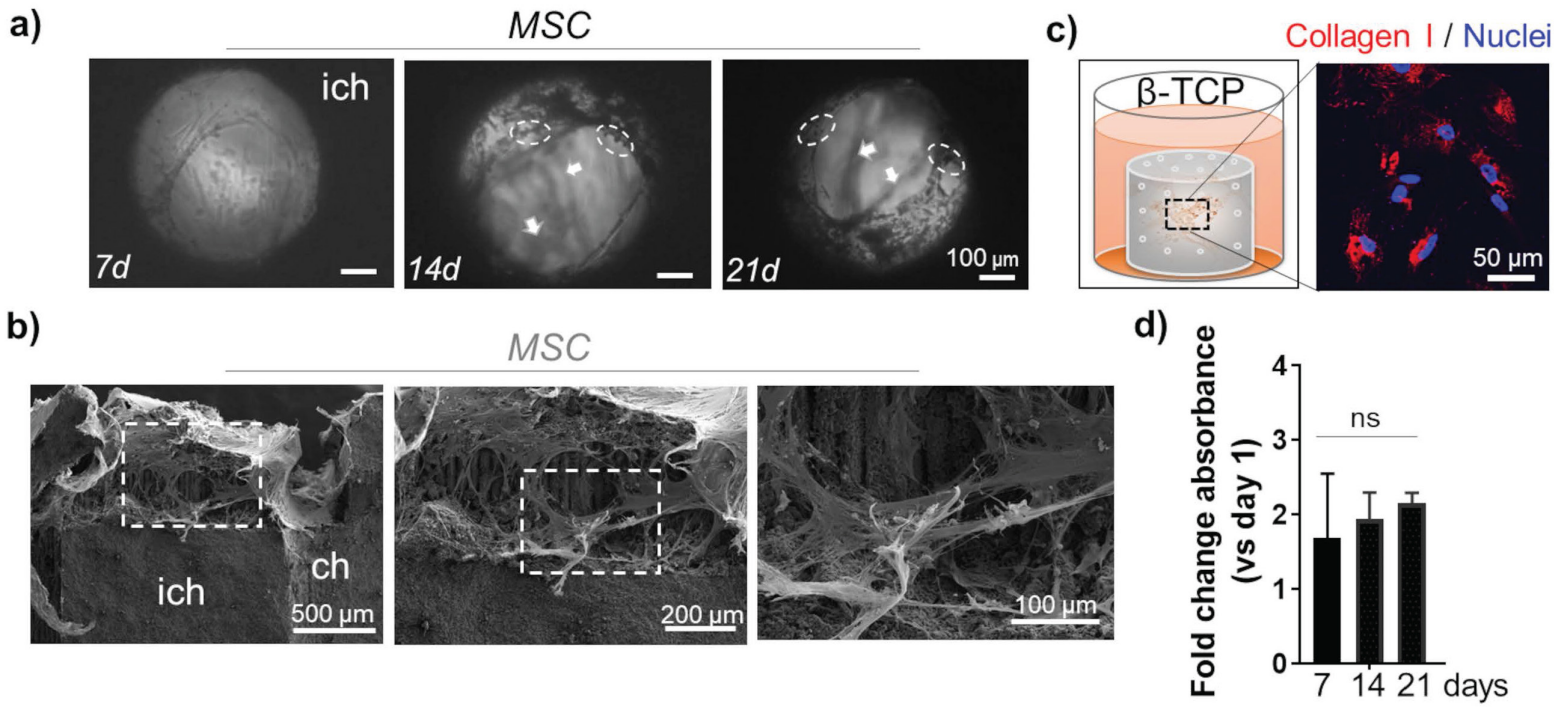
** MSC growth inside the microchannels:** a) Light microscopy: top view inside the interconnected channels. MSC organization after 7d, 14d, and 21d. Arrows indicate the position of different stacks. Dashed circles highlight mineral deposits. Scale bar, 100 μm. ich - inter-channel β-TCP structure. b) SEM images of internal channel surface of the β-TCP scaffolds repopulated with MSC. Scale bar, 100, 200 and 500 μm. c) Evaluation of ECM protein Collagen I (red) production by MSC grown in β-TCP scaffolds. The cartoon depicts the test sample positioning inside a 48 well plate and recalls the cells' organization inside the scaffolding structure's interconnecting channels. DAPI (blue) - nuclear staining. d) Time-dependent MSC proliferation rate in β-TCP scaffolds is presented as a mean fold change respect to day 1 after seeding. ns - not significant.

The morphology of MSC was monitored inside the channels up to 21 days using light microscopy. Sustained cell growth and dense organization in multi-layer structures was confirmed (Figure 2a). As previously reported, a deposition of minerals that is typical for MSC differentiation towards osteoblast-like cells, was apparent in the cells grown in β-TCP scaffolds (Figure 2a).^8^ To ratify that mineralization was triggered by the presence of Ca++, we used a bioinert alumina ceramic with the same 3D geometry as the β-TCP scaffolds. Repeated growth of MSC in alumina scaffolds for 21 days did not have the same effects as β-TCP and no mineral deposits were observed (Supplementary Figure 2a). SEM analysis revealed a homogeneous distribution of MSC in the inner channels of β-TCP, indicating also a strong adhesive interaction between the cells and the microstructure of the scaffold (Figure 2b). The stretching and elongation properties of the MSC within the β-TCP scaffolds were preserved under 3D conditions. The pro-differentiating status of MSC in β-TCP scaffolds that was studied more in details previously 8 was confirmed by evaluating the expression of Collagen I protein. The amount of this ECM protein was abundantly produced by MSC grown in β-TCP (Figure 2c). The observed differentiation of MSC resulted in a time-dependent attenuation of the proliferation rate observed in β-TCP scaffolds (Figure 2d). On the contrary, MSC grown in alumina scaffolds showed no such attenuation after 21 days of *in vitro* culturing (Supplementary Figure 2b) and the production of Collagen I was not comparable to β-TCP (Supplementary Figure 2c).

### 3.3. Cell culturing in 3D microenvironment

**Figure 3:**
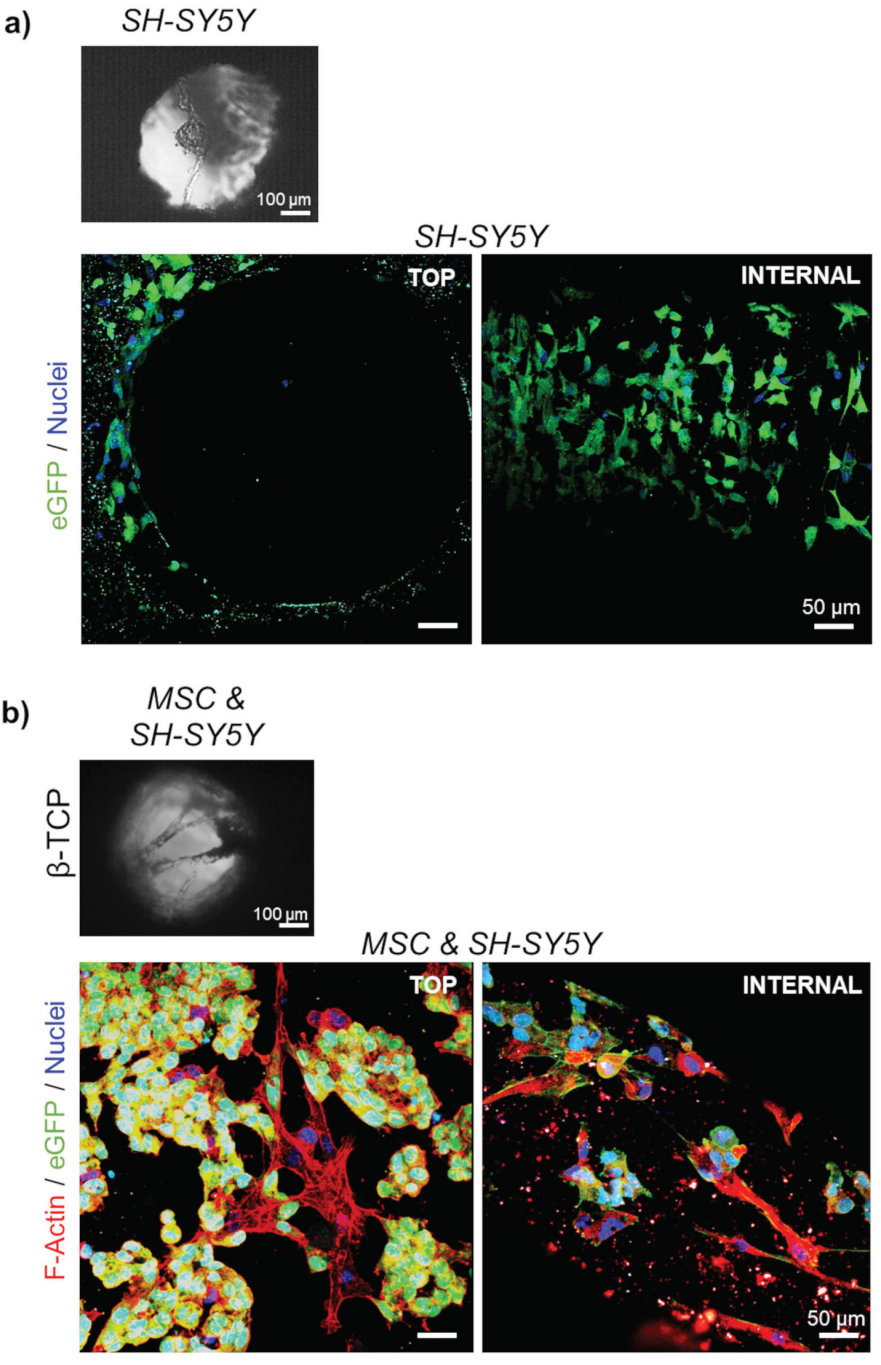
** Organization of the cells in 3D growth condition:** Light microscopy of a) mono-cultured neuroblastoma (SH-SY5Y) cells or b) neuroblastoma cells co-cultured with differentiating MSC for 7 days. SEM micrographs correspond to the top views inside the interconnected channels. Scale bar, 100 μm. Two-photon microscopy images show cell organization - top view and internal investigation (after cutting the scaffolds). Phalloidin (F-Actin) - red; DAPI - cell nuclei (blue); eGFP - neuroblastoma cells (green). Scale bar, 50 μm.

**Figure 4:**
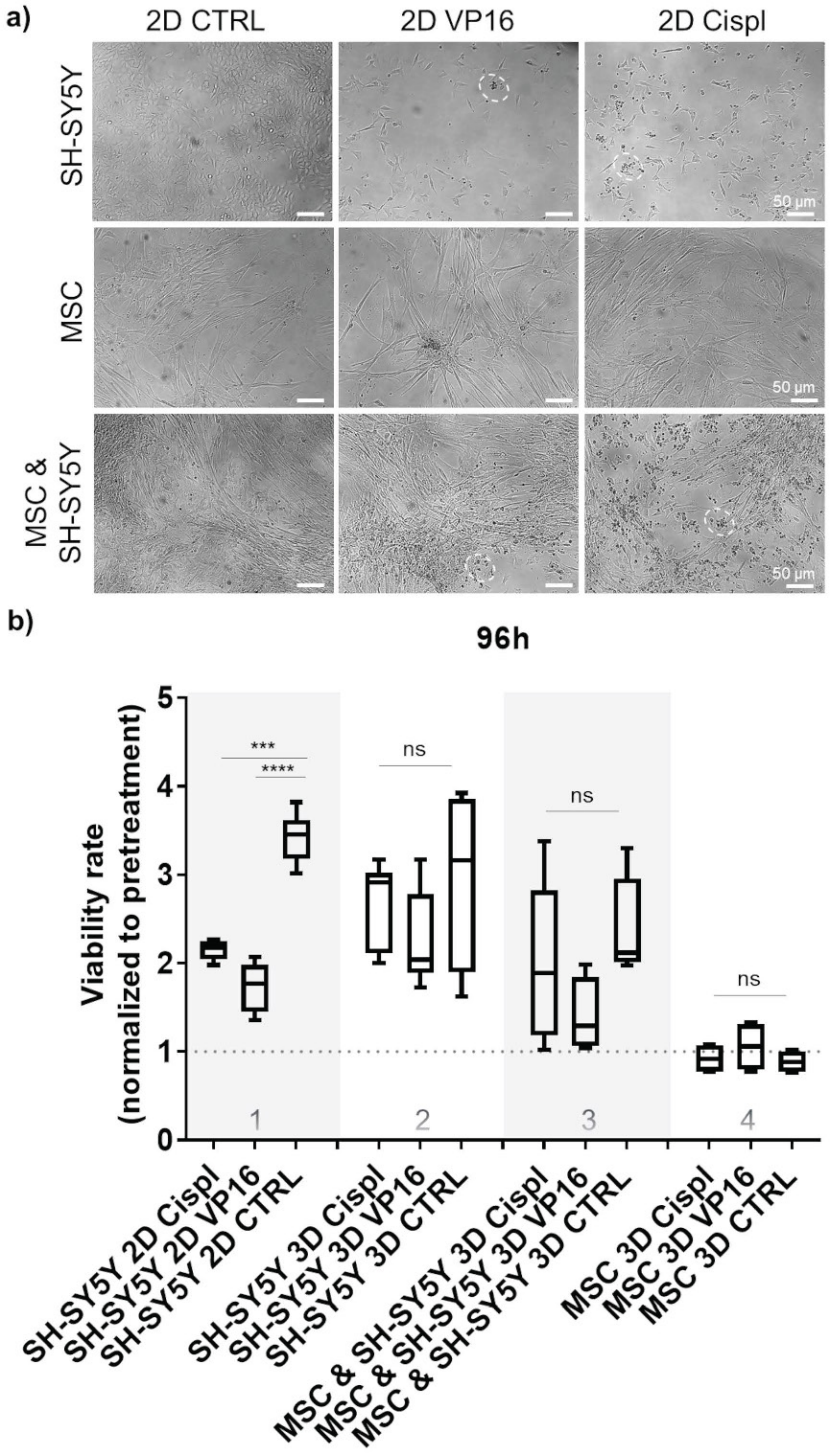
** Effect of chemotherapy drugs on cell viability in 2D and 3D cell systems:** a) Morphology of treated cells grown in 2D as mono-culture (neuroblastoma tumor cells (SH-SY5Y) or MSC alone), and as cell co-culture (MSC and neuroblastoma cells together) with 5 μM of two chemotherapeutics, VP16 (Etoposide) or Cispl (Cisplatin), for 96 hours. Dashed circles highlight the residues of dead tumor cells. Stretched MSC can be observed below the debris of dead tumor cells. MSC isolated from two different donors were considered. b) Viability of treated cells grown in β-TCP scaffolds as cell mono- and co-culture compared to a conventional 2D cell growth. Treatments were done for 96 hours with 5 μM of VP16 (Etoposide) or Cispl (Cisplatin), and viability (1 equal to 100 %) normalized to the values of CCK-8 obtained before adding drugs. CTRL - DMSO treated control cells. ***p<0.001, ****p<0.0001, ns - not significant.

Next, a co-culture of stromal and neuroblastoma cells in β-TCP-3D systems was examined. In the final phase of the experiment, 10732 ± 4087 neuroblastoma cells (18.4 ± 3.3 %; n=3) and 57241 ± 13006 MSC (81.6 ± 3.3 %; n=3) were successfully extracted from a dense 3D cell network and quantified by flow cytometry. Light microscopy showed that neuroblastoma cells grown in single culture did not form an intense reticular structure (Figure 3a and Supplementary Figure 3a) as seen when co-cultured with MSC (Figure 3b and Supplementary Figure 3b). As previously reported, neuroblastoma cells tend to form clusters when grown in 3D β-TCP scaffolds in co-culture with differentiating MSC.^1^,^8^ The previous co-culturing result was replicated with neuroblastoma cells in the form of rosettes (Figure 3b). Moreover, when neuroblastoma cells were seeded alone, they organized macroscopically in the channels as they would under conventional 2D conditions (Figure 3a and 4a).^1^

### 3.4. β-TCP scaffolds as a drug screening platform

To explore the potential of β-TCP scaffolds as a platform for drug screening, we performed a proof-of-principle study to test the effects of conventional chemotherapeutic agents such as VP16 (Etoposide) and Cisplatin (Cispl) on cells in mono- and co-culture. These two drugs are used in current treatment protocols for high-risk neuroblastoma patients with bone-marrow metastatic disease.^27^ To select a concentration range of chemotherapeutic agents that actively inhibits proliferation of two different neuroblastoma cell types (SH-SY5Y and SK-N-BE2) under 2D *in vitro* conditions, a half-maximal inhibitory concentration (IC_50_) was determined (Supplementary Figure 4). The concentration of 5 μM was selected for further studies. At this concentration, the morphology of cells from a mono-culture (neuroblastoma tumor cells or MSC) or from a co-culture (MSC and neuroblastoma cells together) confirmed selective toxicity of chemotherapeutic agents toward neuroblastoma tumor cells and no significant effects on MSC (Figure 4a and Supplementary Figure 5). Thus, MSC maintained a well-stretched, spindle-shaped phenotype in 2D mono- and co-culture. By measuring the cell viability rate in relation to the pretreatment values, we confirmed a good efficacy of the adopted concentrations for each chemotherapeutic agent against neuroblastoma cells grown under 2D conditions (Figure 4b, column 1). However, under 3D conditions, the same drug concentration was not sufficient to replicate the previously observed toxicity rate for neuroblastoma cells (Figure 4b, column 2). Importantly, the introduction of the stromal component (MSC) into the 3D system reduced the differences in viability between the control (CTRL) and chemotherapeutic drug-exposed samples (Figure 4b, column 3), suggesting a protective role of MSC against neuroblastoma cells. Under both conditions, 2D or 3D, a nontoxic effect of the administered drug concentrations on nonmalignant MSC cells was observed (Figure 4b, column 4).

### 3.5. 3D growth condition requires higher dosage of chemotherapeutics

**Figure 5:**
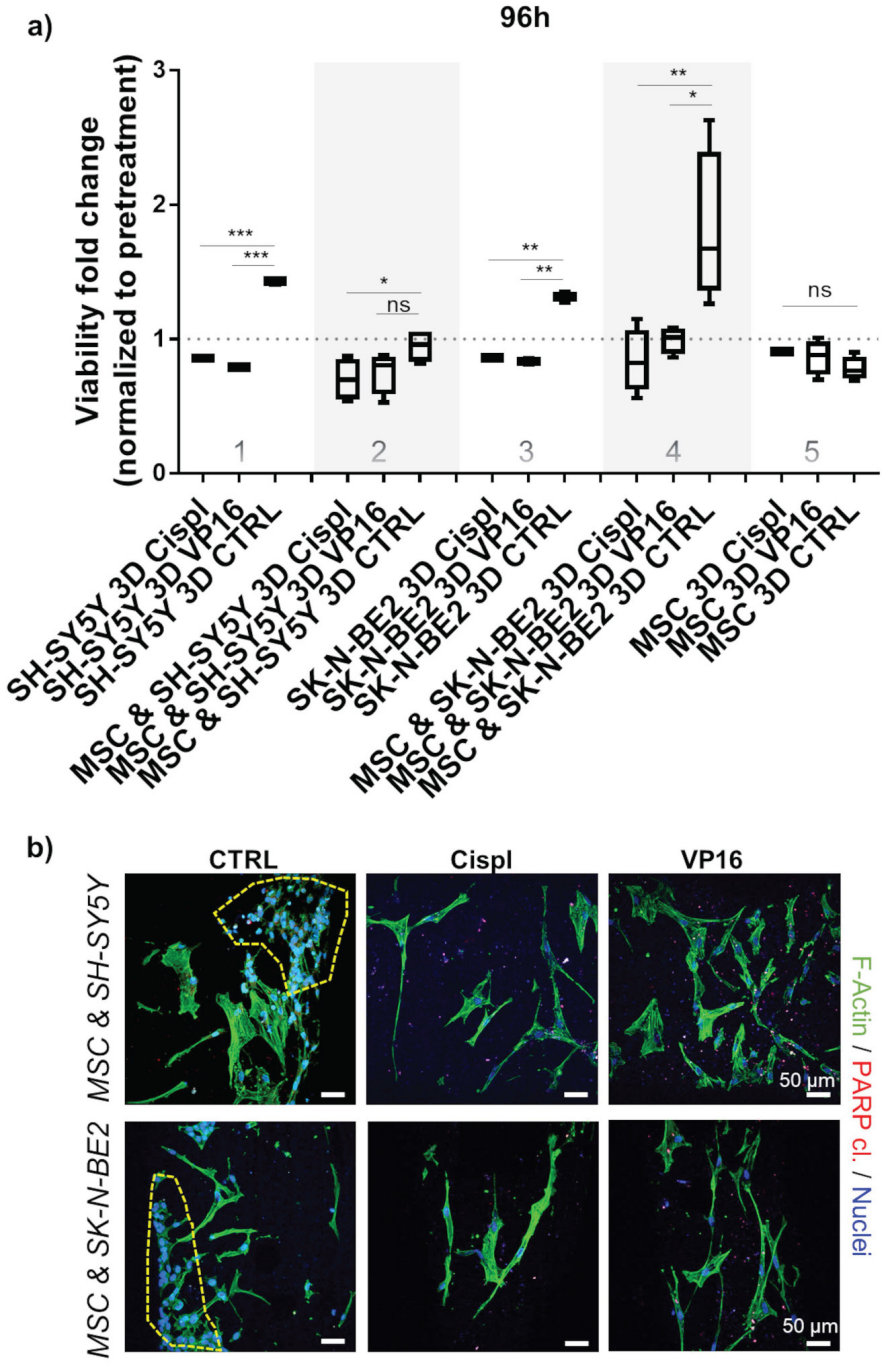
** Chemotherapeutic concentration adjustment under 3D growth:** a) Viability of neuroblastoma cells (SH-SY5Y and SK-N-BE2) was assessed 96 hours post-treatment with 10 μM of VP16 (Etoposide) or Cispl (Cisplatin). Cell mono- and co-cultures were compared. Viability corresponding to 1 (equal to 100 %) presents a normalized values obtained by CCK-8 assay before adding chemotherapeutics. MSC from a new donor were considered. CTRL - DMSO treated control cells. *p<0.05, **p<0.01, ***p<0.001, ns - not significant. b) Two photon microscopy images of 3D co-cultures treated with VP16 or Cispl. DMSO treated samples served as control (CTRL). Internal investigation was performed after cutting the scaffolds. Phalloidin (F-Actin) - green; cleaved PARP protein - residual red puncta; DAPI - cell nuclei (blue). Dashed yellow lines gather major neuroblastoma cell clusters. Scale bar, 50 μm.

To adjust for a discrepancy in the dosage of chemotherapeutics in 2D versus 3D conditions, an independent analysis was performed by increasing the concentrations of VP16 and Cispl from 5 μM to 10 μM. Both neuroblastoma cell types, SH-SY5Y and SK-N-BE2, were challenged in 3D conditions either as a mono-culture or as a co-culture (with MSC). The results confirmed that administration of the increased concentration of chemotherapeutics successfully inhibited the growth of neuroblastoma cells with respect to their controls (Figure 5a, columns 1 and 3). Again, the introduction of MSC into the 3D system increased inter-sample variability and demonstrated that the presence of MSC was critical in protecting neuroblastoma cells from the toxic effects of chemotherapeutic agents (Figure 5a, columns 2 and 4). The increased drug concentration did not significantly affect a growth of the non-malignant stromal cell component of the 3D metastatic bone model (Figure 5a, column 5) confirming a selectivity of administered therapeutics in impeding tumor cell growth. These results were subsequently confirmed qualitatively by immunofluorescence assays. In the co-cultures treated with VP16 or Cispl, preservation of MSC and eradication of the neuroblastoma cell compartment was observed (Figure 5b). In addition, increased residual red puncta, indicative of a late apoptotic death event (cleaved PARP protein), were detected only in the drug treated samples in which neuroblastoma cells were no longer present.

## 4. Discussion

Application of diverse 3D *in vitro* platforms for new drug discovery or drug repurposing is accepted nowadays in pre-clinical pharmaco-oncology as a more versatile approach to select advanced therapeutic strategies. A growing tendency to develop novel *in vitro* experimental tumor models that offer realistically higher translational value compared to 2D cell cultures anticipates a clinical progression of new treatment options as well. Whenever a surgical intervention provides a sufficient amount of tumor material, it is reasonable to attempt the generation of large-scale organoid-based platforms that can be used for automated high-throughput *ex-vivo* drug screenings.^28^,^29^ In cases where this approach is questionable due to a lack of tumor-derived material, alternative strategies need to be adopted in order to obtain biomimetic tumor models. This is the case for brain tumors, for example, but also for many pediatric (solid) malignancies for which there is an urgency for *in vitro* platforms of enhanced physiological relevance suitable for precision medicine and cancer biology assessments. Among these, bioengineering offers valuable *in vitro* options for the reproduction of the complex interactions existing between malignant and non-malignant cellular and acellular components in the neoplastic masses.^30^

Bone metastases of solid tumors cause rapidly progressing disease and even today present a major challenge in clinical oncology. In such cases, conventional chemotherapy protocols are not sufficient to impede malignant cell growth, and hence alternative therapeutics need to be investigated and often within a restricted time frame. In such cases, artificial 3D models of bone metastases can be an option for drug screenings and for prediction of the efficacy that mono- or combined-treatments might have against tumor cells and/or the tumor microenvironment.^31^,^32^ In the case of neuroblastoma, bone metastases occur as a result of chemoresistance and generally lead to a rapid disease evolution.^33^ Due to these factors, the interest in dissecting metastatic neuroblastoma disease at the cellular and molecular level is exploding 34, hence the accessibility of 3D models is playing a determinant role in reaching desirable outcomes.^35^-^37^

The β-TCP scaffolds proposed in this study offer mechanical and biochemical properties of bone, paving the way for a pro-osteogenic differentiation of MSC through calcium phosphate release.^4^,^5^ Tailored scaffolds with interconnected channels sustained the clustering of neuroblastoma cells into an intense reticular structure of MSC. Osteogenic differentiation was sustained in β-TCP 3D configurations by the formation of mineral deposits, which was not observed in alumina scaffolds, but also by an enhanced Collagen I production, confirming previously reported data.^8^,^38^,^39^ These results highlight that 3D geometry defines the cell organization and cell-to-cell connectivity, but that chemistry defines microenvironment specification therefore influencing the performance of different cell types. When challenged as platforms for drug screening, β-TCP scaffolds showed stable readouts between the tested samples. Importantly, neuroblastoma cells grown in 3D β-TCP structures exhibited significant resistance to administered concentrations of chemotherapeutic agents compared with their counterparts treated under 2D conditions. Hence, an increased dosage of VP16 and Cisplatin was requested to trigger tumor cell toxicity in 3D. There are two main reasons why 3D tumor models generally require an increase in chemotherapy concentration: first is the lower diffusion rate, and second refers to an attenuated proliferation rate of cells 1, each making cytotoxic drugs less effective compared to 2D cell systems. These data correlate with the effects of Cisplatin on the growth of other neuroblastoma cell lines (Kelly^Luc^ and Kelly^Cis83Luc^) when cultured in 3D collagen scaffolds.^40^ In that study, the authors showed that Kelly^Cis83Luc^ preserved Cisplatin resistance in 3D culture. Furthermore, Baek et al. treated neuroblastoma cells grown in a 3D spheroid culture system with doxorubicin, confirming an increase in the final IC_50_ values respect with 2D growth conditions.^41^ Our results speak in favor of these reports while settling a discrepancy between effective drug concentrations able to impede growth of the same tumor cell type in 2D and in 3D environment.^42^ However, with respect to those studies, our experimental setting included additional variables, such as stroma cell component and chemical characteristic of the bone microenvironment, to approach more closely the metastatic neuroblastoma bone niche. From this point of view, the proposed 3D model provides an innovative approach for pre-clinical screening of drugs against neuroblastoma. Although there are still numerous challenges to daily use of complex 3D tumor models for routine pre-clinical drug screening, their integration is necessary in order to achieve more restrictive initial selection of functional biologics or personalized therapeutics. In fact, 3D models, not only of neuroblastoma but also of other solid tumors, are being painstakingly used to decipher important molecular and cellular questions relevant to a full understanding of the biological processes underlying these diseases.^16^,^43^,^44^ They all envision the possibility of introducing adequate 3D oncology models for more sophisticated pre-clinical investigations in near future.

## 5. Conclusion

In summary, our results highlight the importance of geometry, chemistry, and cell complexity in defining native-like interaction and connectivity between non-malignant and tumor cells, but also in more realistically determining the efficacy of administered chemotherapy drugs in eradicating metastatic tumor cells in bones.

## Supplementary Material

Supplementary figures.

Supplementary video 1a CT-analysis beta-TCP scaffold.

## References

[1] Aveic S, Janßen S, Nasehi R (2021). A 3D printed in vitro bone model for the assessment of molecular and cellular cues in metastatic neuroblastoma. Biomater Sci [Internet].

[2] Ventura Ferreira MS, Bergmann C, Bodensiek I (2016). An engineered multicomponent bone marrow niche for the recapitulation of hematopoiesis at ectopic transplantation sites. J Hematol Oncol [Internet].

[3] Chamary S, Grenho L, Fernandes MH, Bouchart F, Monteiro FJ, Hornez JC (2021). Influence of a macroporous β-TCP structure on human mesenchymal stem cell proliferation and differentiation in vitro. Open Ceram [Internet].

[4] Yuan H, Fernandes H, Habibovic P (2010). Osteoinductive ceramics as a synthetic alternative to autologous bone grafting. Proc Natl Acad Sci [Internet].

[5] Sen KS, Duarte Campos DF, Köpf M, Blaeser A, Fischer H (2018). The Effect of Addition of Calcium Phosphate Particles to Hydrogel-Based Composite Materials on Stiffness and Differentiation of Mesenchymal Stromal Cells toward Osteogenesis. Adv Healthc Mater [Internet].

[6] Trombetta R, Inzana JA, Schwarz EM, Kates SL, Awad HA (2017). 3D Printing of Calcium Phosphate Ceramics for Bone Tissue Engineering and Drug Delivery. Ann Biomed Eng [Internet].

[7] Ramírez JA, Ospina V, Rozo AA (2019). Influence of geometry on cell proliferation of PLA and alumina scaffolds constructed by additive manufacturing. J Mater Res [Internet].

[8] Aveic S, Davtalab R, Vogt M (2019). Calcium phosphate scaffolds with defined interconnecting channel structure provide a mimetic 3D niche for bone marrow metastasized tumor cell growth. Acta Biomater [Internet].

[9] Chen Z, Li Z, Li J (2019). 3D printing of ceramics: A review. J Eur Ceram Soc [Internet].

[10] Edmondson R, Broglie JJ, Adcock AF, Yang L (2014). Three-Dimensional Cell Culture Systems and Their Applications in Drug Discovery and Cell-Based Biosensors. Assay Drug Dev Technol [Internet].

[11] Monteiro CF, Custódio CA, Mano JF (2019). Three-Dimensional Osteosarcoma Models for Advancing Drug Discovery and Development. Adv Ther.

[12] Yan X, Zhou L, Wu Z (2019). High throughput scaffold-based 3D micro-tumor array for efficient drug screening and chemosensitivity testing. Biomaterials [Internet].

[13] Mapanao AK, Voliani V (2020). Three-dimensional tumor models: Promoting breakthroughs in nanotheranostics translational research. Appl Mater Today [Internet].

[14] Brancato V, Garziano A, Gioiella F (2017). 3D is not enough: Building up a cell instructive microenvironment for tumoral stroma microtissues. Acta Biomater [Internet].

[15] Belloni D, Heltai S, Ponzoni M (2018). Modeling multiple myeloma-bone marrow interactions and response to drugs in a 3D surrogate microenvironment. Haematologica [Internet].

[16] Borella G, Da Ros A, Borile G (2021). Targeting mesenchymal stromal cells plasticity to reroute acute myeloid leukemia course. Blood [Internet].

[17] Ham J, Lever L, Fox M, Reagan MR (2019). In Vitro 3D Cultures to Reproduce the Bone Marrow Niche. JBMR Plus [Internet].

[18] Nasehi R, Abdallah AT, Pantile M (2023). 3D geometry orchestrates the transcriptional landscape of metastatic neuroblastoma cells in a multicellular in vitro bone model. Mater Today Bio [Internet].

[19] Granchi D, Corrias MV, Garaventa A (2011). Neuroblastoma and bone metastases: Clinical significance and prognostic value of Dickkopf 1 plasma levels. Bone.

[20] Brodeur GM (2003). Neuroblastoma: biological insights into a clinical enigma. Nat Rev Cancer [Internet].

[21] Matthay KK, Maris JM, Schleiermacher G (2016). Neuroblastoma. Nat Rev Dis Prim.

[22] Rifatbegovic F, Frech C, Abbasi MR (2018). Neuroblastoma cells undergo transcriptomic alterations upon dissemination into the bone marrow and subsequent tumor progression. Int J Cancer [Internet].

[23] Duarte Campos DF, Bonnin Marquez A, O'Seanain C (2019). Exploring Cancer Cell Behavior In Vitro in Three-Dimensional Multicellular Bioprintable Collagen-Based Hydrogels. Cancers (Basel) [Internet].

[24] Piconi C, Porporati AA (2016). Bioinert Ceramics: Zirconia and Alumina. In: Handbook of Bioceramics and Biocomposites [Internet]. Cham: Springer International Publishing.

[25] Schindelin J, Arganda-Carreras I, Frise E (2012). Fiji: an open-source platform for biological-image analysis. Nat Methods [Internet].

[26] Kasten P, Beyen I, Niemeyer P, Luginbühl R, Bohner M, Richter W (2008). Porosity and pore size of β-tricalcium phosphate scaffold can influence protein production and osteogenic differentiation of human mesenchymal stem cells: An in vitro and in vivo study. Acta Biomater [Internet].

[27] Hara J (2012). Development of treatment strategies for advanced neuroblastoma. Int J Clin Oncol [Internet].

[28] Mainardi S, Bernards R (2022). A large-scale organoid-based screening platform to advance drug repurposing in pancreatic cancer. Cell Genomics [Internet].

[29] Langhans SA (2018). Three-Dimensional in Vitro Cell Culture Models in Drug Discovery and Drug Repositioning. Front Pharmacol [Internet].

[30] Choi SR, Yang Y, Huang KY, Kong HJ, Flick MJ, Han B (2020). Engineering of biomaterials for tumor modeling. Mater Today Adv [Internet].

[31] Jinnah A, Zacks B, Gwam C, Kerr B (2018). Emerging and Established Models of Bone Metastasis. Cancers (Basel) [Internet].

[32] Bock N, Kryza T, Shokoohmand A (2021). In vitro engineering of a bone metastases model allows for study of the effects of antiandrogen therapies in advanced prostate cancer. Sci Adv [Internet].

[33] Garaventa A, Parodi S, De Bernardi B (2009). Outcome of children with neuroblastoma after progression or relapse. A retrospective study of the Italian neuroblastoma registry. Eur J Cancer [Internet].

[34] Lazic D, Kromp F, Rifatbegovic F (2021). Landscape of Bone Marrow Metastasis in Human Neuroblastoma Unraveled by Transcriptomics and Deep Multiplex Imaging. Cancers (Basel) [Internet].

[35] Ornell KJ, Coburn JM (2019). Developing preclinical models of neuroblastoma: driving therapeutic testing. BMC Biomed Eng [Internet].

[36] Yogev O, Almeida GS, Barker KT (2019). In Vivo Modeling of Chemoresistant Neuroblastoma Provides New Insights into Chemorefractory Disease and Metastasis. Cancer Res [Internet].

[37] Corallo D, Frabetti S, Candini O (2020). Emerging Neuroblastoma 3D In Vitro Models for Pre-Clinical Assessments. Front Immunol [Internet].

[38] Vater C, Kasten P, Stiehler M (2011). Culture media for the differentiation of mesenchymal stromal cells. Acta Biomater [Internet].

[39] Rau J V, Fosca M, Fadeeva I V (2020). Tricalcium phosphate cement supplemented with boron nitride nanotubes with enhanced biological properties. Mater Sci Eng C [Internet].

[40] Curtin C, Nolan JC, Conlon R (2018). A physiologically relevant 3D collagen-based scaffold-neuroblastoma cell system exhibits chemosensitivity similar to orthotopic xenograft models. Acta Biomater [Internet].

[41] Baek N, Seo OW, Kim M, Hulme J, An SSA (2016). Monitoring the effects of doxorubicin on 3D-spheroid tumor cells in real-time. Onco Targets Ther [Internet].

[42] Ornell KJ, Mistretta KS, Newman E, Ralston CQ, Coburn JM (2019). Three-Dimensional, Scaffolded Tumor Model to Study Cell-Driven Microenvironment Effects and Therapeutic Responses. ACS Biomater Sci Eng [Internet].

[43] Jakubikova J, Cholujova D, Hideshima T (2016). A novel 3D mesenchymal stem cell model of the multiple myeloma bone marrow niche: biologic and clinical applications. Oncotarget.

[44] Mosaad E, Chambers K, Futrega K, Clements J, Doran MR (2018). Using high throughput microtissue culture to study the difference in prostate cancer cell behavior and drug response in 2D and 3D co-cultures. BMC Cancer.

