# Supplementary material for: Three-dimensional in vitro model of bone metastases of neuroblastoma as a tool for pharmacological evaluations
Source: Nanotheranostics. 2024 Jan 1;8(1):1–11. doi: 10.7150/ntno.85439 (PMC10750120; doi:10.7150/ntno.85439)
Supplement: Supplementary file 1 — Supplementary figures. [file ntnov08p0001s1.pdf]

Supplementary material

Supplementary figure legends

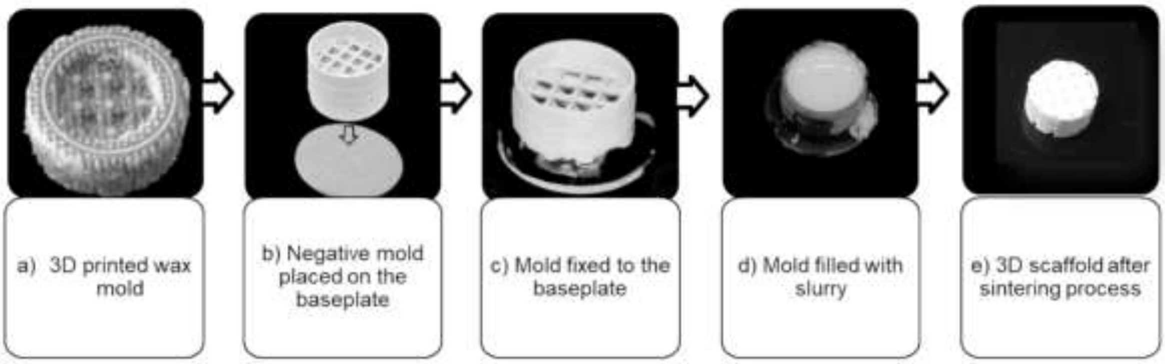

Supplementary Figure 1. Step-by-step workflow of 3D scaffold fabrication.

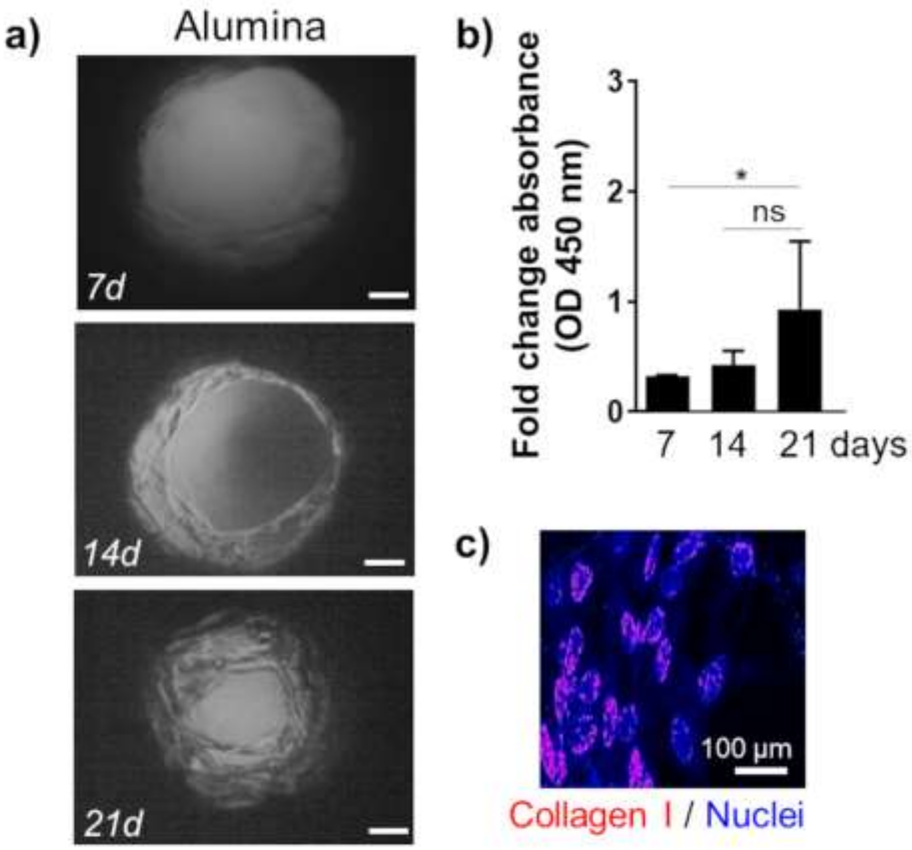

Supplementary Figure 2. MSC growth inside microchannels of alumina scaffolds: a) Light microscopy: top view inside the interconnected channels. MSC organization after 7 days (d), 14d, and 21d. Scale bar, 100 µm. b) Time-dependent MSC proliferation rate in alumina scaffolds is presented as a mean fold change respect to day 1 after seeding. ns – not significant, *p<0.05. c) Evaluation of ECM protein Collagen I (red) production by MSC grown in alumina scaffolds. DAPI (blue) - nuclear staining. Scale bar, 100 µm.

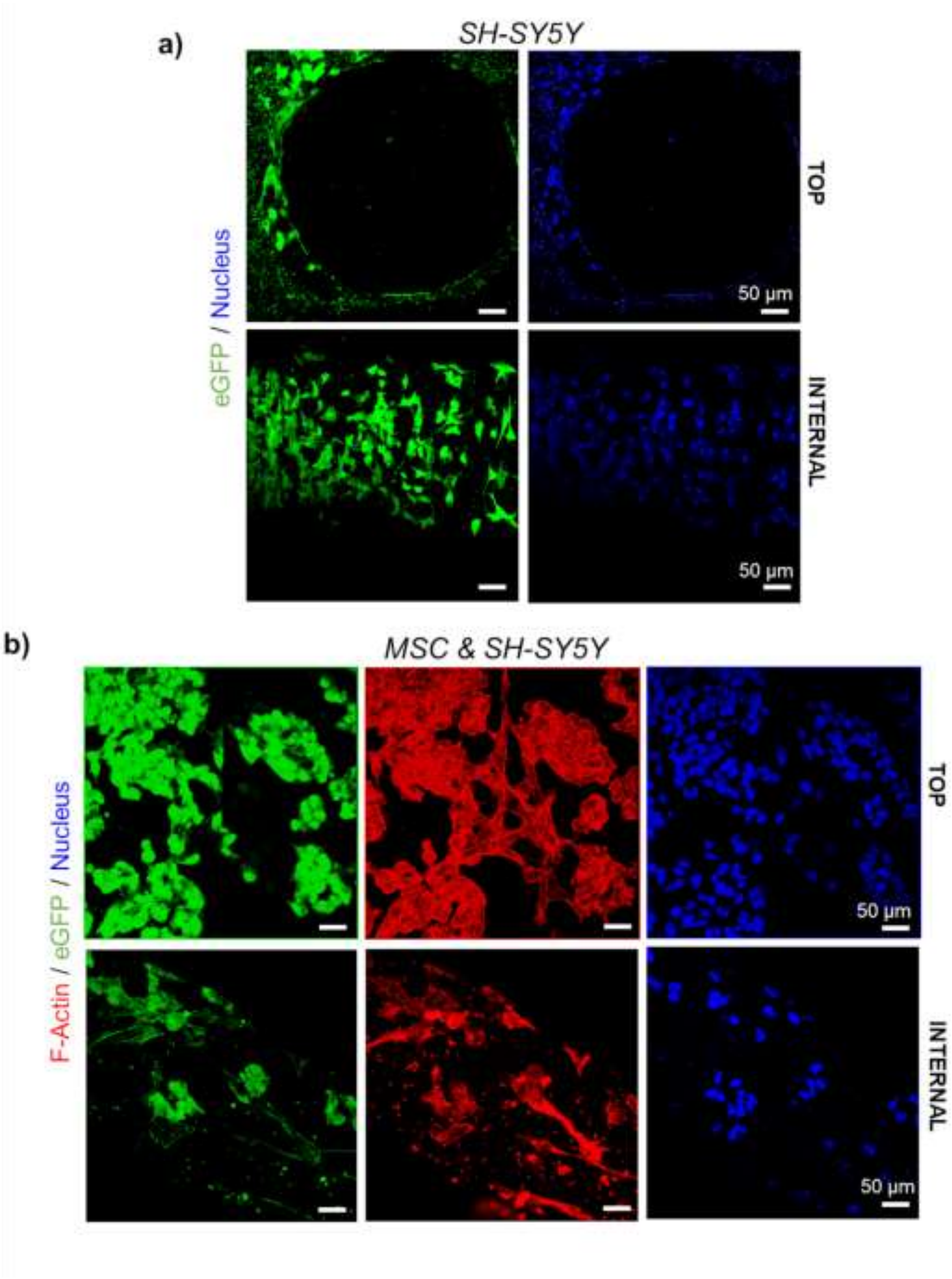

Supplementary Figure 3. Organization of the cells in 3D growth condition: Single channels of the two-photon microscopy images from the main Figure 3. a) Mono-culture of neuroblastoma (SH-SY5Y) cells and b) co-culture of neuroblastoma cells and differentiating MSC for 7 days. The top views and internal side of the scaffold after cutting is shown. Phalloidin (F-Actin) – red; DAPI – cell nuclei (blue); eGFP – neuroblastoma cells (green). Scale bar, 50 µm.

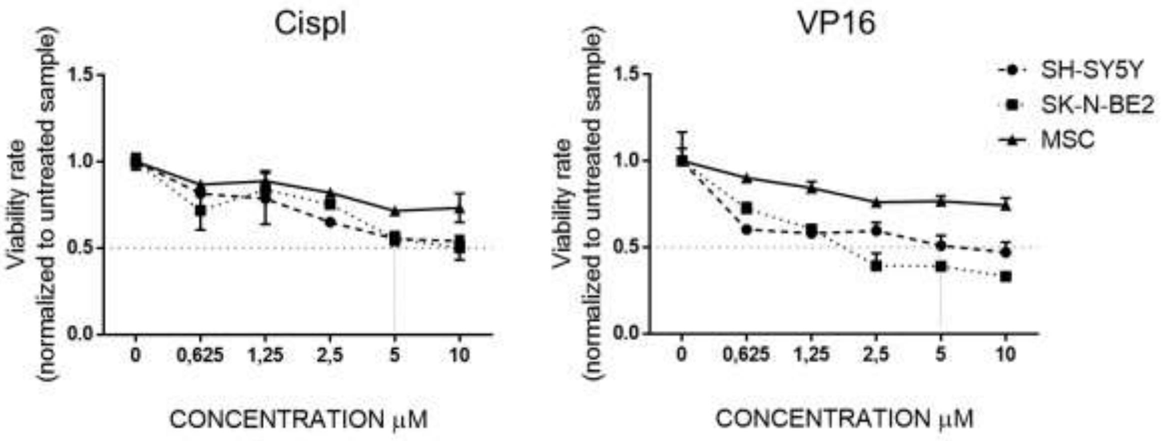

Supplementary Figure 4. A half-maximal inhibitory concentration (IC_{50}) evaluation in 2D. Two neuroblastoma cell lines, SH-SY5Y and SK-N-BE2, and non-malignant MSC were treated with increasing concentration of VP16 (Etoposide) and Cispl (Cisplatin). X axis – drug concentration in µM. Y axis – viability rate determined by CCK8. DMSO treated control sample, corresponding to 0 µM drug concentration, was used for data normalization. Gray dashed line indicates 50% viability rate.

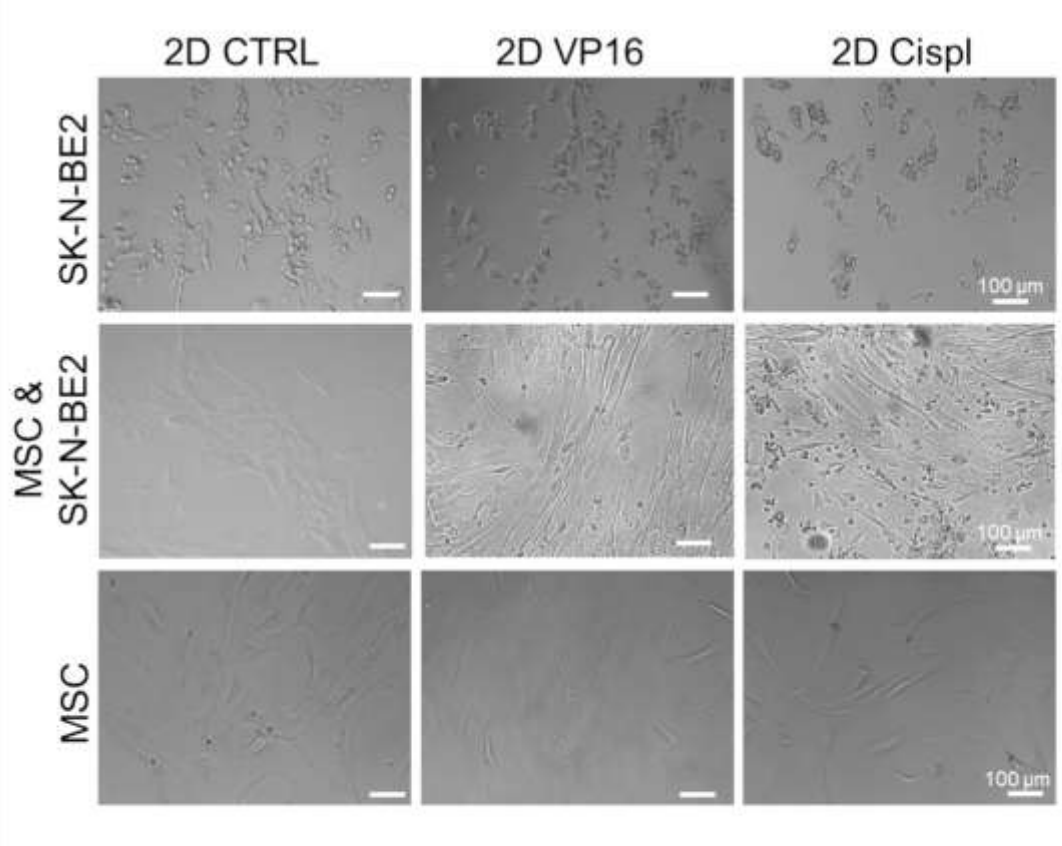

Supplementary Figure 5. Effect of chemotherapy drugs on cell viability in 2D cell systems: Morphology of the cells grown in 2D as mono-culture (neuroblastoma tumor cell line (SK-N-BE2) or MSC) and co-culture (MSC and neuroblastoma) with 5 µM of each chemotherapeutics, VP16 (Etoposide) or Cispl (Cisplatin), for 96 hours.
